# Thyroid bud morphogenesis requires CDC42- and SHROOM3-dependent apical constriction

**DOI:** 10.1242/bio.014415

**Published:** 2016-01-15

**Authors:** David A. F. Loebel, Timothy F. Plageman, Theresa L. Tang, Vanessa J. Jones, Maria Muccioli, Patrick P. L. Tam

**Affiliations:** 1 Embryology Unit, Children's Medical Research Institute, Locked Bag 32, Wentworthville, New South Wales 2145, Australia; 2 Sydney Medical School, University of Sydney, Sydney, New South Wales 2006, Australia; 3 Ohio State University College of Optometry, Columbus, OH 43210-1280, USA

**Keywords:** Endoderm, Foregut, Thyroid, Organogenesis, Apical constriction, Cell polarity, Epithelial, Organ bud

## Abstract

Early development of the gut endoderm and its subsequent remodeling for the formation of organ buds are accompanied by changes to epithelial cell shape and polarity. Members of the Rho-related family of small GTPases and their interacting proteins play multiple roles in regulating epithelial morphogenesis. In this study we examined the role of *Cdc42* in foregut development and organ bud formation. Ablation of *Cdc42* in post-gastrulation mouse embryos resulted in a loss of apical-basal cell polarity and columnar epithelial morphology in the ventral pharyngeal endoderm, in conjunction with a loss of apical localization of the known CDC42 effector protein PARD6B. Cell viability but not proliferation in the foregut endoderm was impaired. Outgrowth of the liver, lung and thyroid buds was severely curtailed in *Cdc42*-deficient embryos. In particular, the thyroid bud epithelium did not display the apical constriction that normally occurs concurrently with the outgrowth of the bud into the underlying mesenchyme. SHROOM3, a protein that interacts with Rho GTPases and promotes apical constriction, was strongly expressed in the thyroid bud and its sub-cellular localization was disrupted in *Cdc42*-deficient embryos. In *Shroom3* gene trap mutant embryos, the thyroid bud epithelium showed no apical constriction, while the bud continued to grow and protruded into the foregut lumen. Our findings indicate that *Cdc42* is required for epithelial polarity and organization in the endoderm and for apical constriction in the thyroid bud. It is possible that the function of CDC42 is partly mediated by SHROOM3.

## Abstract (precis)

**Summary:** Conditional *Cdc42* knockout revealed requirements for *Cdc42* in endoderm polarity, and in thyroid apical constriction and morphogenesis. *Shroom3* mutant embryos also displayed thyroid bud abnormalities, suggesting a possible functional interaction.

## INTRODUCTION

In mouse embryos, the epithelial cell layer of the primitive gut tube is derived from the definitive endoderm, which is generated from the epiblast. During gastrulation, cells fated for the definitive endoderm emerge from the primitive streak, transit through the mesoderm layer and intercalate into the pre-existing layer of visceral endoderm (Kwon et al., 2008; Viotti et al., 2014). Allocation of epiblast cells to the definitive endoderm is completed by the early-somite stage. Consequently, morphogenesis of the gut and associated organs is sustained by the expansion of a finite pool of epiblast-derived progenitor cells (Loebel et al., 2014b). The maintenance of cell viability and proliferation of this population requires the activity of the transcription factor SOX17 (Kanai-Azuma et al., 2002). At the early organogenesis stage, the endoderm layer becomes internalized, beginning with the formation of the foregut and hindgut invaginations and the progressive closure of the midgut to form a complete tube. In the foregut invagination, the epithelial cells lining its ventral and lateral walls adopt a polarized columnar morphology (Loebel and Tam, 2012). Concurrently with gut tube morphogenesis, the primordia of the thyroid, liver, lungs and pancreas are formed in the foregut as local outgrowths of organ buds (Loebel et al., 2014b). The formation of organ buds begins with the localized re-modeling of the epithelium, including a thickening of the endoderm and rearrangement of the epithelial cells, which in the case of the thyroid and liver involves the formation of a multi-layered epithelium (Loebel et al., 2014b).

Members of the Rho family of small GTPases play multiple roles in the morphogenesis and re-modeling of epithelia. In mouse embryos in which *Rhou* was knocked down, the foregut endoderm lost its columnar epithelial architecture, lacked apical accumulation of F-actin and displayed fewer actin-rich microvilli at the apical surface (Loebel et al., 2011). Consistent with this, overexpression of *Rhou* alters F-actin distribution and junction formation in epithelial cells *in vitro*, whereas knockdown of *Rhou* severely impairs the formation of lumen-containing epithelial cysts (Brady et al., 2009). In mouse embryos, foregut endoderm cells, in which *Rhou* was knocked down, disengaged from the apical domain of the epithelium and retracted to the basal region. This cellular behavior is reminiscent of initial stages of multilayering that occurs at the prospective site of organ bud formation coinciding with the down-regulation of *Rhou* (Loebel et al., 2011; Loebel and Tam, 2012).

The role of another Rho-related GTPase, CDC42, in epithelial morphogenesis has been extensively studied in cell culture. CDC42 interacts with the PDZ-domain containing protein PARD6B and atypical protein kinase C (aPKC) to promote apical junction formation (Joberty et al., 2000). Knockdown of *Pard6b* or *Prkz* (encoding aPKC) in cultured epithelial cells has similar effects to knockdown of *Cdc42* (Durgan et al., 2011), and the interaction between CDC42 and PAR6 is required for establishing epithelial polarity (Hutterer et al., 2004). Expression of constitutively active or dominant negative forms of CDC42 disrupts both the polarity of epithelial cell and cyst formation (Rogers et al., 2003). Knocking down *Cdc42* alters spindle pole orientation, positioning of the cellular apical domains and results in the formation of epithelial cysts with multiple lumens (Bray et al., 2011; Jaffe et al., 2008). In *Drosophila* embryos, CDC42 promotes epithelial polarity, influences cytoskeletal organization and changes cell shape during lumen formation (Eaton et al., 1995; Genova et al., 2000).

In mice, *Cdc42* is critical for the morphogenesis of epithelial structures during development. Mouse embryos lacking *Cdc42* do not develop past implantation and do not form an epithelial epiblast (Chen et al., 2000) and embryoid bodies derived from *Cdc42*-null embryonic stem cells display defective epithelial polarity and cell junctions (Wu et al., 2007). Later in development, *Cdc42* is required for establishing cell polarity and initiating tubule formation in the pancreas and for aligning cellular polarity and spindle pole orientation in lung epithelia during branching morphogenesis (Kesavan et al., 2009; Wan et al., 2013). However, the roles of *Cdc42* earlier in the development of the gut endoderm and organ buds have not been examined.

The thyroid bud is the first recognizable organ bud to form. The epithelial placode of the early bud in the midline ventral endoderm is marked by expression of *Nkx2-1*, *Foxe1*, *Pax8* and *Hhex* (Fagman and Nilsson, 2010). Initially, cells of the thyroid placode are taller than surrounding cells and adopt a pseudostratified organization, subsequently forming a multi-layered primordium that protrudes into the underlying mesenchyme. Cells of the thyroid bud retain epithelial cell characteristics, such as the expression of E-cadherin and the decoration of cell junctions by β-catenin, indicating that no epithelial-mesenchymal transition has occurred at this stage (Fagman et al., 2003). Prior to detachment from the endoderm, the thyroid bud grows in size but cells contained within the bud are not highly proliferative. It has been shown in chick embryos that the cell number in the bud increases by the recruitment of endoderm cells from outside the bud (Fagman et al., 2006; Smuts et al., 1978). By embryonic day (E)11.5-12.5, the thyroid bud becomes detached from the foregut and is displaced towards the trachea.

In this study, we examined the requirement for *Cdc42* in the morphogenesis of the foregut endoderm and organ bud formation. By conditionally ablating *Cdc42* with a tamoxifen-inducible CRE recombinase, we showed that *Cdc42* is required for the maintenance of cell shape and polarity in the foregut endoderm and for initiating the outgrowth of organ buds. Apical constriction and epithelial bending in the thyroid bud, which are necessary for expansion into the underlying mesenchyme, are impaired by the loss of *Cdc42* activity. SHROOM3, a PDZ-domain-containing protein that interacts with Rho GTPases in regulating cell shape, was mislocalized in CDC42-deficient cells. Loss of function of *Shroom3* also impaired epithelial bending but not the overall growth of the thyroid bud, suggesting that the apical constriction that is required for normal morphogenesis of the thyroid bud is dependent on CDC42 and SHROOM3 activity.

## RESULTS

### Impact of conditional ablation of *Cdc42*

Constitutive loss of *Cdc42* function (complete knockout) results in a failure of epiblast development and an inability to progress beyond implantation (Chen et al., 2000). To overcome the confounding effect of early embryonic lethality, a tamoxifen-inducible *Cre* transgene, *Ubc*-CreERT2, was used (Loebel et al., 2014a; Ruzankina et al., 2007) to control the time of ablation of *Cdc42* in embryos harboring conditional *Cdc42*-flox alleles (Wu et al., 2006) during post-implantation development to investigate the impact of *Cdc42* deficiency on foregut morphogenesis. To test the efficacy of CRE recombinase in deleting a target sequence, *Cdc42^flox/+^; Ubc*-CreERT2 mice were crossed with ROSA26R reporter mice and tamoxifen administered to the pregnant female at E5.5 (*n*=2 embryos sectioned), E6.5 (*n*=3) or E8.5 (*n*=4). Staining for β-galactosidase reporter activity revealed widespread but incomplete CRE-mediated excision in the endoderm and other tissues of the embryo irrespective of the stage of tamoxifen administration (Fig. S1A-E).

To determine the optimal timing of induction of CRE activity for the analysis of gut morphogenesis and early organogenesis, we first tested the effects of tamoxifen treatment at E5.5, E6.5 and E7.5. *Cdc42^flox/+^; Ubc*-CreERT2 mice were crossed with *Cdc42^flox/flox^* mice to generate CKO (*Cdc42^flox/flox^; Ubc*-CreERT2) and control (CRE negative) embryos. When pregnant mice were given oil only (vehicle control), embryos developed normally regardless of genotype (Fig. S2A,B). Following tamoxifen administration at E5.5, embryos were collected at E7.75 and E8.5. Conditional knockout (CKO) embryos were consistently smaller than controls and appeared to have ceased developing prior to E8.5 (Fig. S2C-F). Sectioning of E7.75 embryos revealed a trilaminar morphology in the CKO embryos as in the controls, indicating that CKO embryos had commenced gastrulation (*n*=3; Fig. S2G,H). However, whereas the endoderm of control embryos displayed a squamous morphology that is characteristic of the definitive endoderm at this stage (Loebel and Tam, 2012) (*n*=2; Fig. S2G), the endoderm of CKO embryos inappropriately retained the tall vacuolated epithelial phenotype of the extraembryonic visceral endoderm of control embryos (Fig. S2H,I). CKO embryos collected at E8.5 from mothers given tamoxifen at E6.5 were also smaller than control embryos (Fig. S2J,K) and, unlike control embryos, did not form a foregut invagination. Whereas the ventral foregut endoderm of control embryos had adopted a characteristic columnar appearance, endoderm in the equivalent location in CKO embryos was thin and disorganized (*n*=3; Fig. S2L,M). Administration of tamoxifen at E7.5 resulted in CKO embryos that were smaller at E9.5 than control embryos, had not commenced axis turning and displayed a truncated head. Whereas by E9.5, embryos normally have a closed gut tube and organ bud development has commenced (Fig. S2N,P,R), CKO embryos developed a rudimentary foregut pocket, but other parts of the prospective gut remained open and there was no sign of organ bud formation in the foregut (Fig. S2O,Q,S).

Since the phenotypic effects of tamoxifen administration before or during gastrulation were too catastrophic to allow a proper examination of endoderm or organ bud development, we next administered tamoxifen at E8.5, during early organogenesis and collected embryos at E9.5 and E10.5. Genotyping of yolk sacs confirmed that deletion of the flox allele was detectable by 24 h after tamoxifen administration (Fig. S1F). Consistent with the reporter staining experiments, the flox allele was still detectable at 24 and 48 h after tamoxifen was given, indicating incomplete excision of the conditional allele (Fig. S1F,G). Immunostaining for CDC42 protein revealed a weaker signal in CKO embryos than in the control (Fig. S1H,I).

**Fig. 1.**
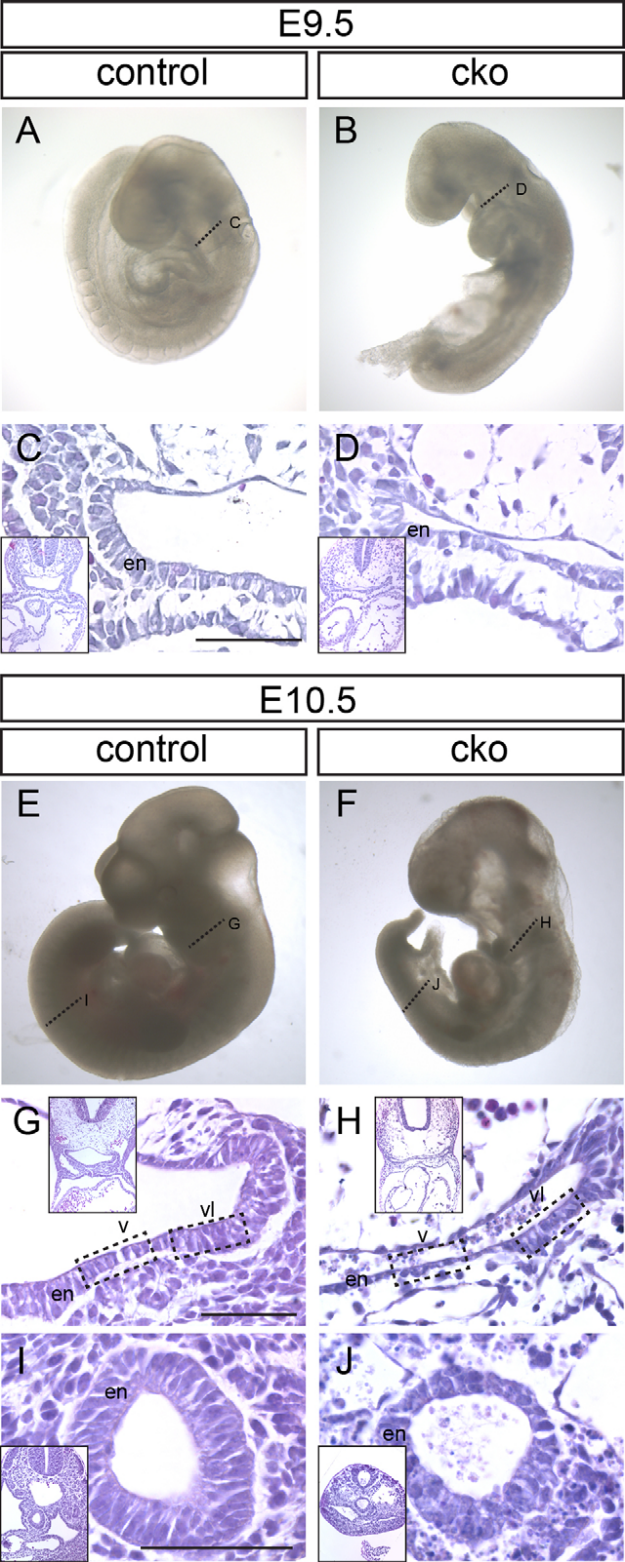
**Consequences of *Cdc42* ablation following tamoxifen administration at E8.5.** (A-D) Control (A) and *Cdc42*-conditional knockout (cko; B) embryos from the same litter collected at E9.5. (C,D) Sections through the foregut (fg) region of embryos at the locations indicated by the dotted lines in panels A and B. (E-J) Control (E) and conditional knockout (F) embryos collected at E10.5. Dotted lines indicate the location of sections through the foregut (G,H) and hindgut (I,J) regions (hg). Boxed regions in G and H indicate the ventral (v) and ventral-lateral (vl) foregut endoderm (en) referred to in subsequent figures. Low magnification images corresponding to each panel are shown as insets. Scale bar=100 μm.

CKO embryos were smaller than wild-type or heterozygous littermates (*n*=12 CKO embryos, *n*=40 for other genotypes, 10 litters analyzed). In particular, the trunk and tail regions were shorter despite that CKO embryos developed similar numbers of somites as controls, suggesting that the rate of development was not affected but the overall tissue mass might have been reduced (Fig. 1A,B,E,F). The ventral endoderm in the pharyngeal region of E9.5 and E10.5 control embryos displayed the expected columnar epithelial morphology whereas, at E9.5, the CKO foregut was dorsoventrally compressed and had a deflated lumen. The ventral endoderm, whilst still consisting primarily of a single cell layer, appeared uneven in thickness and the cells were irregularly organized (Fig. 1C,D). At E10.5, the contrast between control and CKO was more pronounced. The ventral endoderm was notably thinner in the CKO, whereas the lateral endoderm was less severely affected (Fig. 1G,H). In the hindgut region, the endoderm layer was also thinner in CKO than in control embryos and the epithelial organization was disrupted (Fig. 1I,J), albeit to a lesser extent than that of the ventral wall of the foregut.

We tested whether ablation of CDC42 affected cell proliferation or survival in the foregut endoderm. We detected no significant difference in cell proliferation between control and CKO embryos, as indicated by the relative proportion of Ki67-positive cells in the endoderm (Fig. S3A). Some cells within the foregut endoderm were positive for cleaved Caspase 3, and clumps of cleaved Caspase 3 positive cells were present in the foregut lumen, suggesting that some apoptotic cells were dislodged from the foregut epithelium and accumulated in the lumen (*n*=3; Fig. S3B,C).

### Altered epithelial architecture in pharyngeal endoderm

**Fig. 2.**
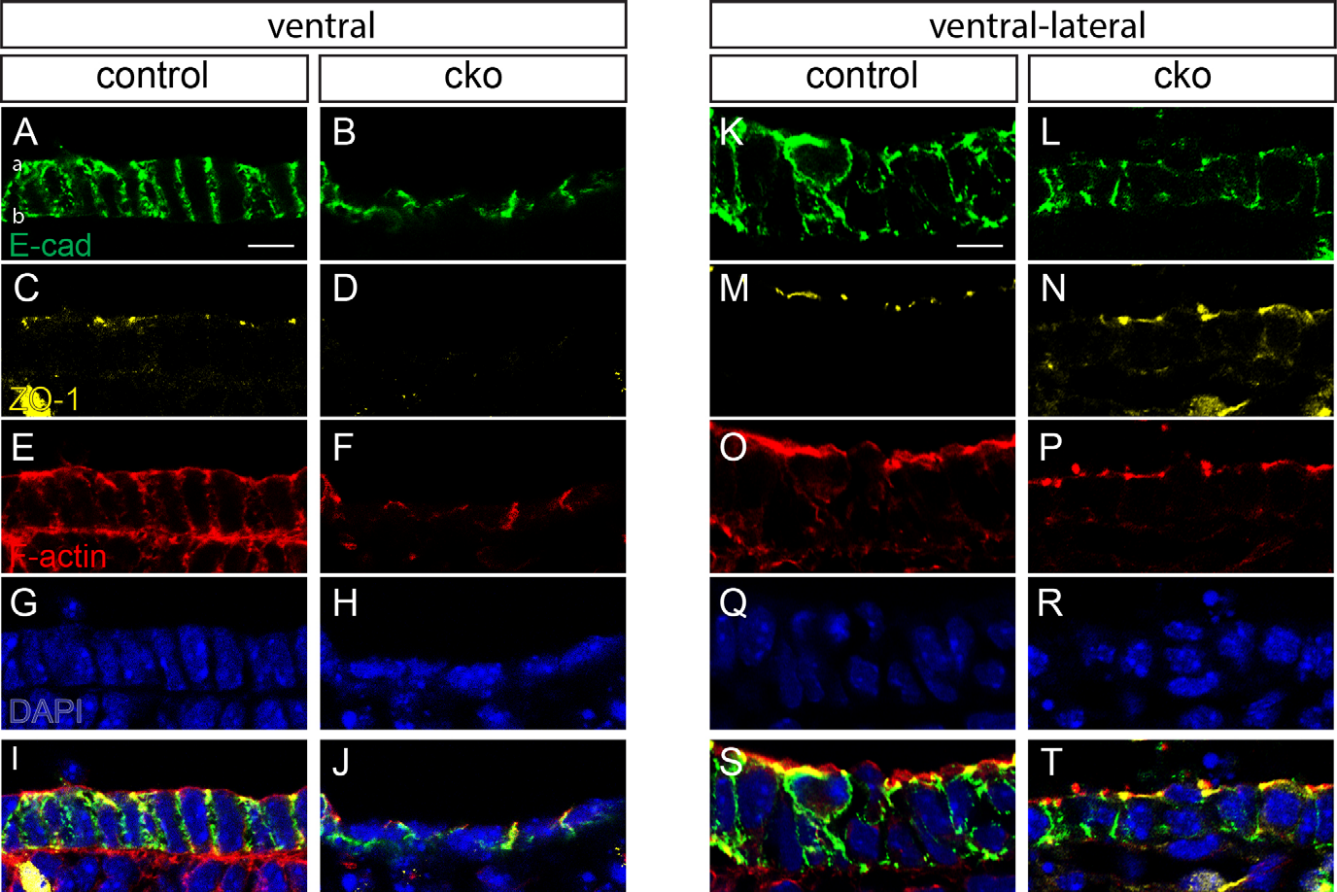
**Disrupted foregut endodermal cell shape and polarity in conditional knockout embryos.** Immunofluorescent staining of ventral foregut (A-J) and ventral lateral foregut (K-T) of E10.5 control (A,C,E,G,I,K,M,O,Q,S) and conditional knockout (cko; B,D,F,H,J,L,N,P,R,T) embryos for E-cadherin (A,B,K,L), ZO-1 (C,D,M,N), staining of F-actin with phalloidin (E,F,O,P) and nuclei with DAPI (G,H,Q,R). Apical and basal aspects of the endoderm are indicated by ‘a’ and ‘b’, respectively. Scale bar=10 μm.

We further investigated the tissue architecture of the ventral and lateral endoderm in the foregut by immunofluorescence for a series of epithelial and cell polarity markers. E-cadherin is a component of adherens junctions, present at the intercellular borders and aligned parallel to the apicobasal axis of the cell in control embryos (Fig. 2A). In the endoderm layer of CKO embryos the columnar morphology was lost and E-cadherin was distributed in an irregular pattern (*n*=4; Fig. 2B). ZO-1 (TJP1), which is normally present in apical tight junctions (Fig. 2C), was reduced in CKO embryos and virtually undetectable in the ventral endoderm of three out of four specimens (Fig. 2D). F-actin, concentrated near the apical cell surface as well as distributed widely in the cortical region in the endoderm of control embryos (Fig. 2E), was confined to the intercellular interface in CKO embryos (Fig. 2F). Fluorescence measurements revealed a reduced apical:basal ratio of F-actin staining intensity in the CKO embryo: CKO 2.195±0.043 vs control 7.183±0.794, (mean±s.e.m.; *P*=0.003 by two-tailed *t*-test, *n*=3; Fig. 2I,J). Differences between control and CKO embryos were less pronounced in the ventral-lateral endoderm (Fig. 2K-T). E-cadherin staining of CKO embryos indicated a more modest reduction in the thickness of the endoderm layer than that of the ventral endoderm (Fig. 2K,L vs Fig. 2A,B). The reduction in ZO-1 (Fig. 2M,N) and F-actin staining in the apical region of the epithelium was also less pronounced (Fig. 2O-T).

**Fig. 3.**
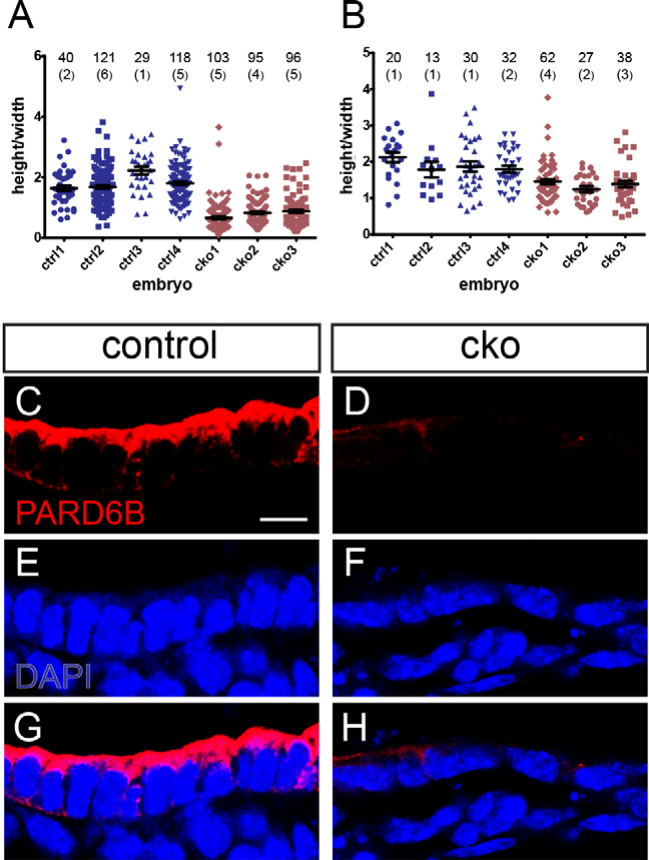
**Altered nuclear shape and loss of apical PARD6B staining in conditional knockout embryos.** (A,B) Ratio of nuclear height to width in the ventral (A) and ventral lateral (B) endoderm of four control (ctrl) and three conditional knockout (cko) embryos. The numbers of cells measured per embryo and the number of sections (in parentheses) are indicated on the plots. (C-H) Immunofluorescent staining for PARD6B and counterstaining with DAPI in the ventral endoderm of control (C,E,G) and conditional knockout (D,F,H) embryos. Scale bar=10 μm.

To quantify the difference in cell shape between control and CKO embryos, we measured the ratio of nucleus height (perpendicular to apical surface) to width (parallel to apical surface, Fig. 3A,B). Whereas in both the ventral and lateral endoderm, the height of nuclei was approximately 1.8 times the width in control embryos, nuclei were wider than they were high in the ventral endoderm of CKO embryos, (mean±s.e.m.; control= 1.83±0.14, *n*=4; CKO=0.79±0.07, *n*=3; Fig. 3A,B). In the ventral-lateral endoderm, the height:width ratio was significantly reduced in CKOs but not to the same extent as in the ventral endoderm (mean±s.e.m.; control =1.85±0.04; CKO =1.33±0.04).

PARD6B is a known effector of CDC42 in epithelial cells and functions in the establishment of apical-basal polarity and the formation of tight junctions (Joberty et al., 2000). In control embryos, PARD6B in the foregut endoderm was apically localized in ventral foregut endoderm cells (*n*=2; Fig. 3C,E,G). In CKO embryos, apical PARD6B staining was greatly reduced in the thinner epithelial cell layer of all specimens analyzed (*n*=3; Fig. 3D,F,H). This observation is consistent with the previously reported reduction in *Pard6b* expression in *Cdc42*-deficient intestinal epithelium (Melendez et al., 2013). Together these data demonstrate that CDC42 is required for the foregut endoderm to maintain its normal columnar morphology, including cytoskeletal polarity, cell shape and apical junction formation. Our data are consistent with CDC42 acting via PARD6B, a known CDC42 effector and a component of the apical polarity machinery.

### Dorso-ventral tissue patterning is unaffected by Cdc42 loss of function but organ bud formation is impaired

**Fig. 4.**
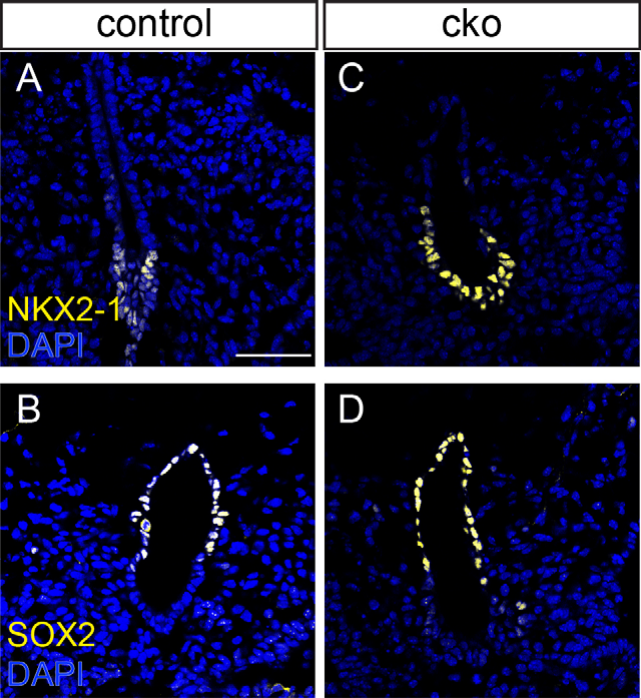
**Dorsal-ventral patterning of the foregut endoderm.** Immunostaining for the ventral endoderm marker NKX2-1 (A,C) and dorsal marker SOX2 (B,D) in the endoderm of control (A,B) and conditional KO (C,D) embryos at E10.5. Scale bar=100 μm.

A likely phenomenon associated with the loss of columnar epithelial architecture in the ventral foregut endoderm is the loss of ventral identity, and/or the dorsalization of the ventral endoderm. In both control and CKO embryos, NKX2-1 marked the ventral endoderm and SOX2 was expressed in the dorsal endoderm (Fig. 4). These data indicate that dorsal-ventral patterning of the embryonic foregut is unaffected in *Cdc42* CKO embryos (*n*=2).

**Fig. 5.**
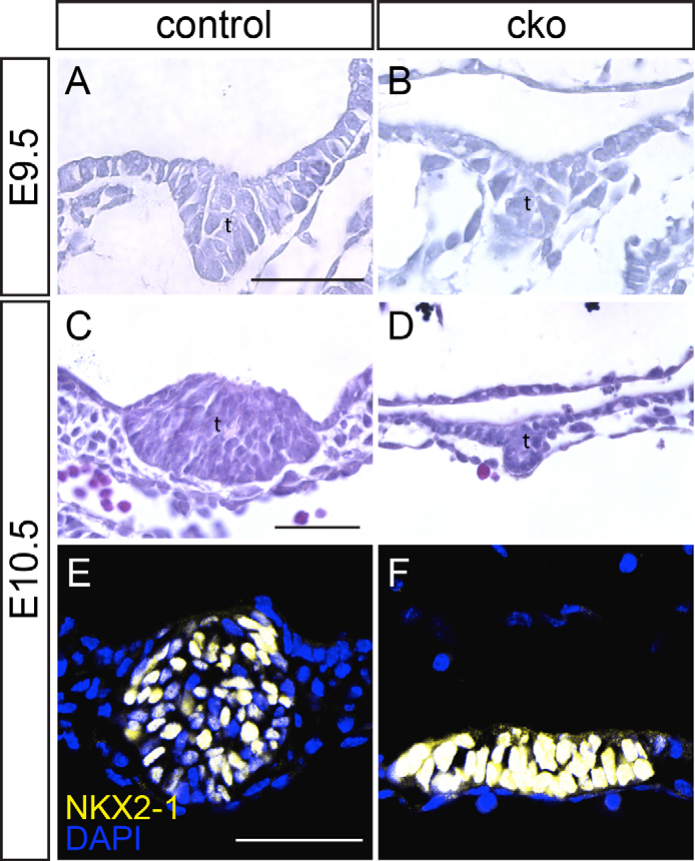
**Impaired thyroid development following CDC42 ablation.** (A-D) Sections through the thyroid bud of control (A,C) and conditional knockout (cko; B,D) embryos at E9.5 and E10.5 (t, thyroid bud). (E,F) Immunofluorescent staining for NKX2-1 in the thyroid buds of control and conditional knockout embryos. Scale bar=100 μm.

We next examined the formation of the organ buds from the CDC42-deficient foregut. At E9.5, the CKO liver bud was small compared with that of a control embryo (Fig. S4A,B). At E10.5, hepatoblasts in the expanding liver primordium of control embryos could be distinguished histologically by their greater density and more intense staining than surrounding cells, and by E-cadherin staining (Fig. S4C,E). By contrast, the liver bud of CKO embryos failed to expand into the surrounding mesenchyme and E-cadherin positive hepatoblasts were absent (control, *n*=3; CKO, *n*=3; Fig. S4D,F). Lung bud development at E10.5 was also impaired in CKO embryos, with nascent buds being either underdeveloped or absent in the tissue adjacent to the NKX2-1-positive domain in the ventral endoderm (Fig. S4G-K). The thyroid bud was smaller in the CKO than in the control embryo at E9.5 (Fig. 5A,B). By E10.5, the thyroid bud was clearly underdeveloped in CKO embryos compared with control embryos (Fig. 5C-F; *n*=8 control, *n*=9 CKO, 3 litters analyzed). In E10.5 control embryos, the thyroid bud was shaped into a multilayered epithelial structure in the floor of the foregut (Fig. 5C), or as a tightly packed cellular clump with a small central lumen that bulged into the subjacent mesenchyme (Fig. 5E), prior to detaching from the epithelium and being displaced ventrally. In CKO embryos, the thyroid buds displayed reduced multi-layering and formed either a small outgrowth (Fig. 5D), or remained as a flat multilayered epithelial patch in the ventral endoderm (Fig. 5F). NKX2-1 was detected in both a control and a CKO thyroid bud, confirming that the bud cells were correctly specified (Fig. 5E,F).

### Impaired epithelial bending and thyroid bud outgrowth in CKO embryos

**Fig. 6.**
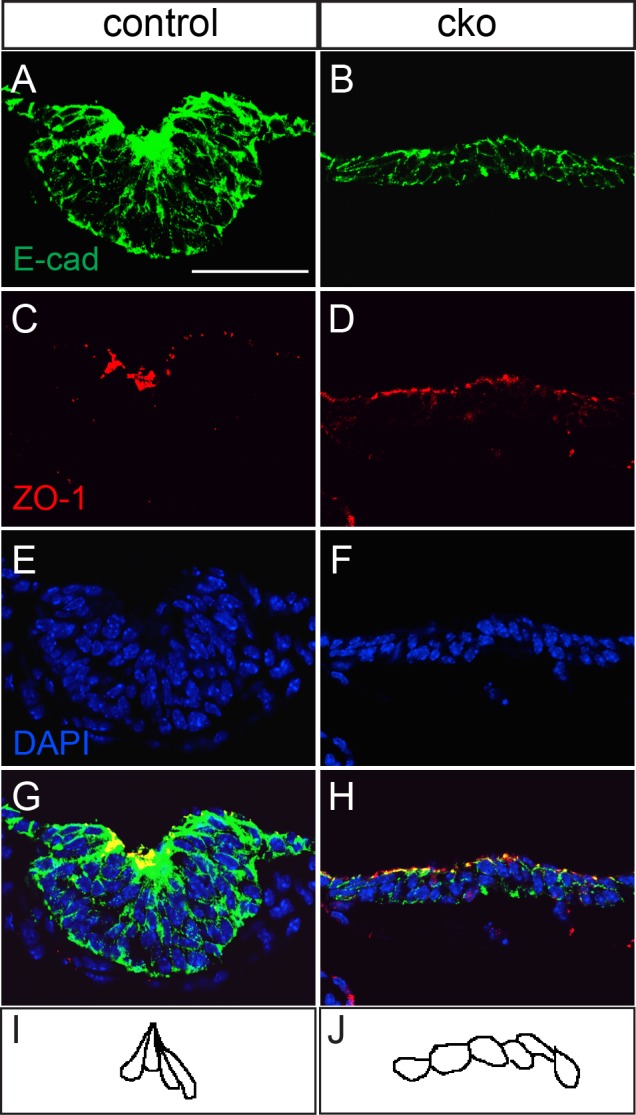
**Lack of epithelial bending in *Cdc42*-deficient thyroid buds at E10.5.** (A-H) Immunostaining of thyroid buds from control (A,C,E,G) and conditional knockout (cko; B,D,F,H) thyroid buds for E-cadherin (A,B) and ZO-1 (C,D), with nuclei counterstained with DAPI (E,H), merged in (G,H). (I) Tracing of the cell outlines at the point of epithelial bending in the thyroid bud from a control embryo. (J) Trace of the cell outlines in the central portion of the unbent epithelium of the thyroid bud from a conditional knockout embryo. Scale bar=100 μm.

Immunostaining revealed an accumulation of E-cadherin and ZO-1 at the junctions of cells at the point of greatest curvature of epithelium of the thyroid bud of control embryos (*n*=3; Fig. 6A,C). Cells at the point of bending in the thyroid bud were narrower apically than at the basal aspect (Fig. 6E,G,I), which is indicative of apical constriction, a mechanism known to drive epithelial bending and tissue morphogenesis (Sawyer et al., 2010). E-cadherin and ZO-1 were widely distributed in the thyroid primordium of the CKO embryos and showed no sign of focal concentration (*n*=3; Fig. 6B,D). No apical constriction was found in CKO thyroid buds (Fig. 6F,H,J).

**Fig. 7.**
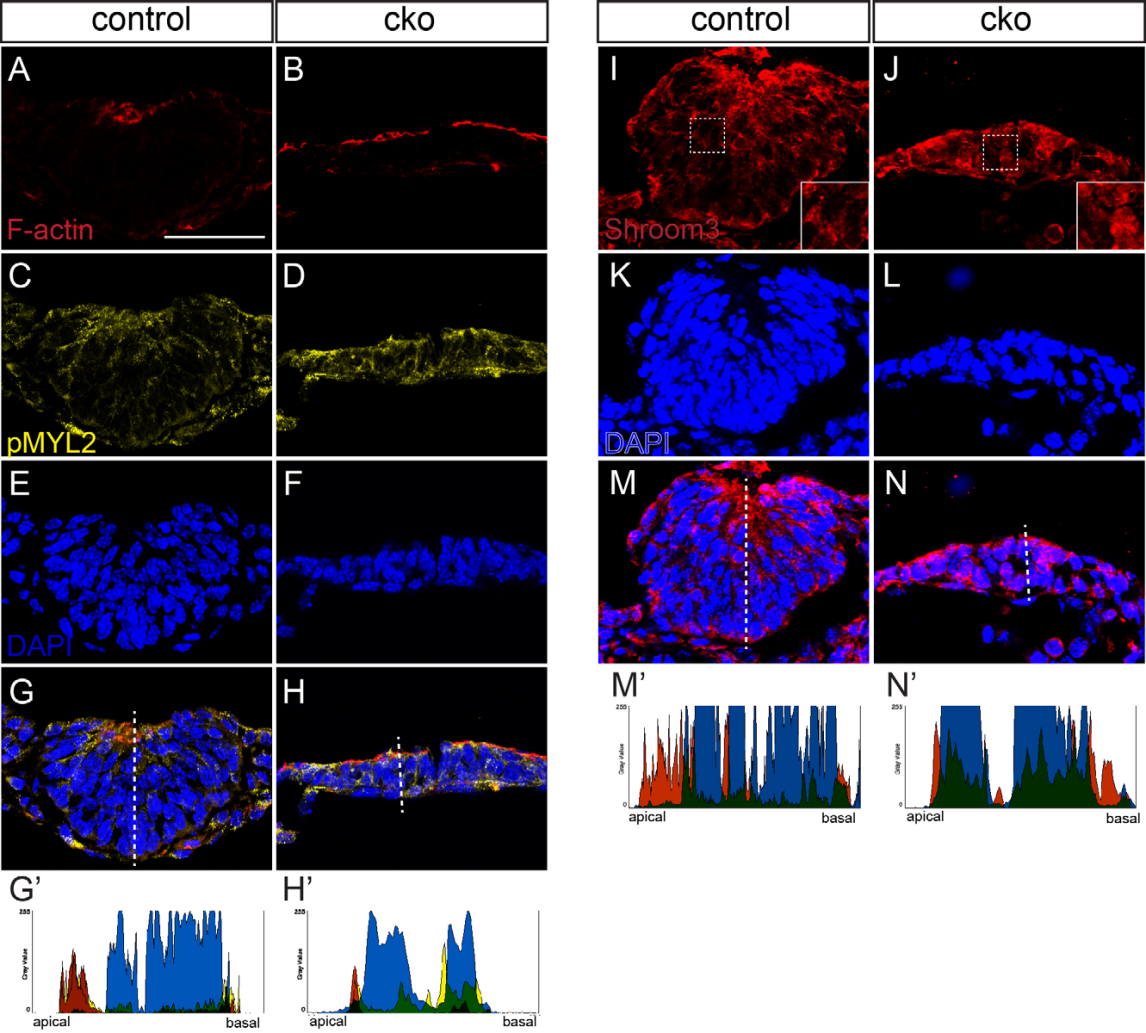
**Impaired apical constriction in *Cdc42*-deficient thyroid buds.** (A-H) Phalloidin staining of F-actin (A,B) and immunostaining of phosphorylated myosin II light chain (C,D) in control and conditional knockout (cko) thyroid buds. (I-N) Immunostaining for SHROOM3 in control and conditional knockout thyroid buds. Insets in I and J show higher magnification of the corresponding boxed region in the main panel. Specimens were counterstained with DAPI to reveal the nuclei (E,F,K,L). (G-H′,M-N′). Labelling was merged in G,H,M,N, and fluorescence intensity was measured on the representative specimen shown in G′,H′,M′,N′, in the plane of the dotted lines in panels G,H,M,N. Colors shown in the charts indicate the protein stained with the same color in the corresponding fluorescence image.

Apical constriction is often associated with the accumulation of F-actin at the apical surface of the epithelium. Contraction of the apical F-actin is mediated by myosin II. In the thyroid bud of the control embryo, F-actin and phosphorylated (active) myosin II light chain (MYL2) accumulated apically in the region of epithelial bending (Fig. 7A,C,E,G,G′). In the thyroid primordium of CKO embryos, F-actin accumulation could be detected at the apical surface (Fig. 7B,H′). However, although phosphorylated myosin light chain was present, there was no clear apical accumulation and the regions of strongest staining did not overlap with F-actin staining (*n*=3 embryos; Fig. 7B,D,F,H,H′). These findings suggest that the lack of activation of myosin II in the apical domain of the cell may underlie the absence of epithelial bending and the ventral outgrowth of the thyroid bud.

The PDZ domain-containing protein SHROOM3 is necessary for apical constriction and proper epithelial bending in neural tube closure, lens placode development and in the gut mesentery (Hildebrand and Soriano, 1999; Plageman, et al., 2010, 2011) of mouse embryo. Strong SHROOM3 expression was detected by β-galactosidase reporter staining (Fig. S5) in the thyroid bud of *Shroom3* gene-trap embryos (Hildebrand and Soriano, 1999). Immunostaining for SHROOM3 confirmed that it is expressed in the thyroid bud and that its distribution overlaps with F-actin (Fig. S6). Like F-actin, SHROOM3 was concentrated at the apical region of the thyroid bud and staining was particularly strong at the point of epithelial bending (Fig. S6B,D,F). We examined SHROOM3 protein localization in the thyroid bud of control and CKO embryos (control, *n*=2; CKO, *n*=1). In control embryos, SHROOM3 was detected at the apical aspect of the thyroid bud cells facing the emerging lumen and in the cell peripheries, where peaks of staining intensity did not coincide with strong DAPI staining (Fig. 7I,K,M,M′). In the CKO thyroid bud, SHROOM3 staining was seen throughout the cells (Fig. 7J,L,N,N′). These findings suggest that CDC42 is required for the proper apical localization of factors that mediate apical constriction in the thyroid bud.

### SHROOM3 is required for epithelial bending in the thyroid bud

**Fig. 8.**
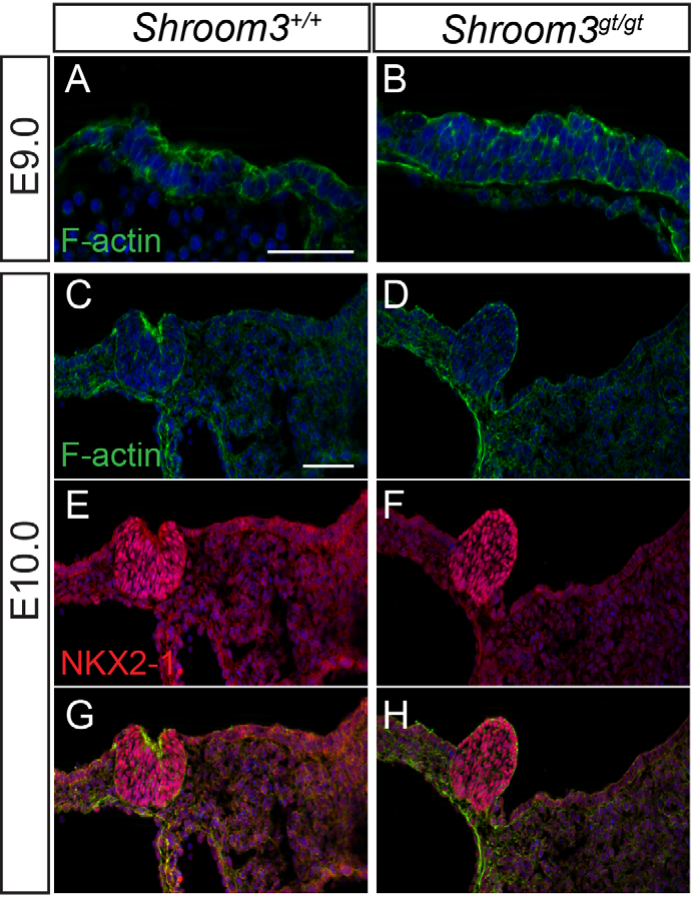
**Early thyroid bud development is abnormal in *Shroom3* deficient embryos.** Immunofluorescently labeled cryosections through the developing thyroid of control and *Shroom3^Gt/Gt^* embryos at the indicated embryonic ages. Sections were labeled with phalloidin to detect F-actin (A-D,G,H), or immunostaining for NKX2-1 (E-H). Scale bars=100 µm.

At E9.0, a multi-layered thyroid placode was present in both control and *Shroom3^Gt/Gt^* embryos. Multi-layering appeared slightly more advanced but more disorganized in *Shroom3^Gt/Gt^* embryos (*n*=3; Fig. 8A,B). By E9.5, the thyroid bud in *Shroom3^Gt/Gt^* embryos had begun to expand into the foregut lumen, rather than downward into the underlying mesoderm, and by E10.0 the abnormality in thyroid development was striking (Fig. 8C-J). Whereas the control thyroid bud had undergone an epithelial bending event and formed a cup shaped structure, the *Shroom3^Gt/Gt^* thyroid bud was dome-shaped and protruded dorsally in all mutant embryos examined (*n*=7; Fig. 8E,F) demonstrating that SHROOM3-dependent apical constriction is necessary for thyroid invagination. We also observed a protruding thyroid bud in all homozygous embryos (*n*=10) that harbored a mutation in the ROCK-binding domain of SHROOM3 (*Shroom3^R1838C^*, Fig. S6G) (Marean et al., 2011). Unlike in *Cdc42* CKO embryos, the bud continued to expand in *Shroom3^Gt/Gt^* mutants and was of a comparable overall size to control thyroid buds. Like *Cdc42* CKO thyroid buds, apical F-actin was detected, but it was not focused at a point of epithelial bending (Fig. 8A,B,E,F,I,J). Both control and *Shroom3^Gt/Gt^* thyroid buds expressed the thyroid-specific marker NKX2-1 (Fig. 8G-J), confirming that in both *Cdc42* CKO and *Shroom3^Gt/Gt^* embryos, the thyroid primordium has been correctly specified.

## DISCUSSION

### CDC42 is required for foregut endoderm cell shape and polarity and may act via PARD6B

In this study we have demonstrated that CDC42 is required for the epithelial morphogenesis of the foregut endoderm. Constitutive loss of *Cdc42* results in embryos that fail to progress beyond implantation and lack an epiblast (Chen et al., 2000). When *Cdc42* is ablated shortly after implantation (E5.5), embryos can proceed through gastrulation and generate a mesoderm-like mesenchymal layer. However, the endoderm cells retain the features of the extraembryonic visceral endoderm, suggesting that the formation of definitive endoderm has been impeded. Ablating *Cdc42* activity later, after the onset of gastrulation, allows for the formation of definitive endoderm-like tissue, but the overall embryonic development is severely impaired, precluding a proper analysis of foregut morphogenesis.

**Fig. 9.**
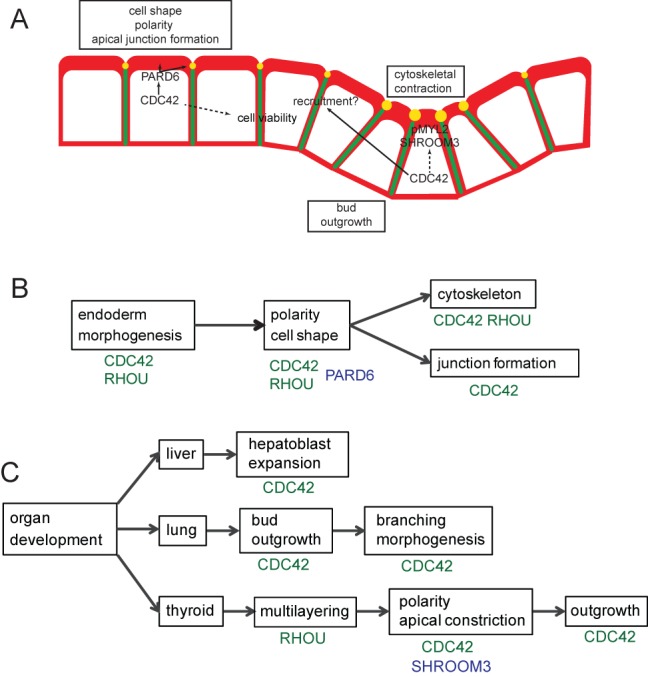
**A working model of the roles of Rho GTPases in endodermal polarity and organ bud development.** (A) In the ventral endoderm CDC42 is required for apical PARD6B, which interacts with CDC42 to control cell shape, polarity (including apical F-actin accumulation), junction formation and cell viability. Within the thyroid bud, CDC42 is necessary for the apical accumulation of SHROOM3 and activated (phosphorylated) non-muscle myosin II, which promote the cytoskeletal contraction necessary for apical constriction and downward growth of the bud. The dotted line indicates that it is not clear whether this is via a direct interaction or through effects on cell polarity. SHROOM3 is required for thyroid bud apical constriction, but CDC42 is also necessary for expansion of the bud. Lack of bud expansion in CDC42 deficient embryos may be due to the structural abnormality of the adjacent endoderm cells that impedes their recruitment to the thyroid bud. F-actin (red), adherens junctions (green) and tight junctions (yellow). (B) *Cdc42* and *Rhou* (Loebel et al., 2011) are both required for proper endoderm morphogenesis and affect cell polarity and shape. This may be due to an interaction with PARD6B. CDC42 and RHOU both affect the actin cytoskeleton, but CDC42 plays an additional role in apical junction formation. (C) CDC42 plays a role in expansion of hepatoblasts in the liver bud and for growth of the lung bud. Later in development, CDC42 influences branching morphogenesis in the lung. In the thyroid, down-regulation of *Rhou* coincides with the onset of multi-layering (Loebel et al., 2011) and CDC42, acting via SHROOM3, affects polarized protein distribution and apical constriction. CDC42 is further required for growth of the bud.

Deletion of *Cdc42* after gastrulation and formation of the foregut allowed us to investigate the requirement for *Cdc42* in the morphogenesis of the gut endoderm and organ buds. Our findings showed that, in *Cdc42* CKO embryos, ventral foregut endoderm cells did not display the columnar epithelial appearance found in control embryos, but instead showed a flattened profile similar to the dorsal foregut endoderm. *Cdc42*-deficient endoderm cells had a reduced apical accumulation of F-actin that was also observed in *Rhou*-deficient foregut endoderm (Loebel et al., 2011). However, loss of *Cdc42* function resulted in more severe polarity defects including reduced apical ZO-1 and PARD6B staining, indicating that the formation of tight junctions was also impaired. Taken together, these data indicate that Rho-related GTPases play overlapping, but not identical roles in endoderm columnar epithelial morphogenesis, with RHOU and CDC42 both influencing the distribution of the actin cytoskeleton and CDC42 playing a more prominent role in apical-basal polarity including affecting the formation of apical cell junctions (Fig. 9A,B).

CDC42 interacts with orthologues of *C. elegans* PAR6 and atypical protein kinase C (aPKC) to regulate the apical cytoskeleton and junction formation. In cultured mammalian epithelial cells, CDC42 resides within a complex including PARD6B, PARD3 and aPKC. CDC42 and PARD6B are both required for the maturation of apical cell junctions (Wallace et al., 2010) and PARD6B must also interact with PARD3 to influence tight junction formation (Joberty et al., 2000). In *Drosophila* embryos, epithelial polarity cannot be established when the cells express a mutant form of PAR6 that is unable to interact with CDC42 (Hutterer et al., 2004). In the present study, deletion of CDC42 resulted in the loss of apical PARD6B and the apical junction marker ZO-1 in the ventral endoderm. This is consistent with the finding that apical localization of PAR6 in Drosophila embryos requires an interaction with CDC42 (Hutterer et al., 2004) and that PARD6B expression is reduced in *Cdc42*-deficient mouse intestinal epithelium (Melendez et al., 2013). Together, these data suggest that a CDC42/PARD6B-containing complex influences apical-basal polarity in the foregut endoderm (Fig. 9A,B).

### CDC42 is required for endodermal organ growth and morphogenesis

In addition to the abnormality in endodermal architecture, we also observed impaired outgrowth of liver, lung and thyroid buds in *Cdc42* CKO embryos. Although we consider that this is most likely to be a result of a primary defect in the endoderm, we cannot exclude that the lack of organ expansion could be mediated in part of a loss of a CDC42-dependent signal from the underlying mesenchymal cells, since *Cdc42* has been ablated throughout the embryo. Previous studies have implicated CDC42 in specific aspects of organ morphogenesis at later stages of development, such as branching morphogenesis in lungs (Wan et al., 2013) and the formation of tubules in the pancreas (Kesavan et al., 2009), which influence cellular differentiation. Our data point to *Cdc42* playing an important role in the early stages of organ bud formation (Fig. 9A,C). In the liver, we observed that initial out-pocketing and multi-layering of the bud occurs, but the liver bud fails to fully invade the surrounding mesenchymal tissues. Multi-layering may involve the downregulation of *Rhou* to trigger cytoskeletal changes that cause cells to alter their position relative to the apical surface (Loebel et al., 2011, Fig. 9C). Lung budding in the NKX2-1-expressing endoderm of CKO embryos was either not visible or severely retarded compared with controls. This phenotypic variability may be due to variations in the rate of development of embryos, relative to the timing of tamoxifen administration.

We have shown that *Cdc42* is also required for proper thyroid bud expansion. Whereas the abnormal *Shroom3*-deficient thyroid buds are able to continue to expand *Cdc42*-CKO thyroid buds were consistently smaller than control buds at the stages examined. The early thyroid bud does not expand by increased cell proliferation, but by recruiting cells from the surrounding endoderm (Fagman et al., 2006; Hilfer et al., 1990; Smuts et al., 1978). The defects in expansion of the thyroid and other organ buds in *Cdc42*-CKO embryos could be partially accounted for by increased cell death in the endoderm. The thyroid bud abnormality might further be exacerbated by the impaired epithelial architecture of the foregut endoderm rendering the cells less competent for incorporation into the thyroid bud (Fig. 9A).

### Thyroid development requires CDC42 and SHROOM3-dependent apical constriction

Apical constriction is a morphogenetic mechanism that promotes bending of epithelial tissue and is integral to branching morphogenesis of the lungs (Kim et al., 2013). In this study we show that the bending of the foregut endoderm, integral to the formation of the thyroid bud, displays the hallmarks of apical constriction, including cell shape changes (narrowing of the apical area) and the apical accumulation of F-actin, phosphorylated myosin light chain II and SHROOM3. Apical constriction is an ATP dependent process and can be inhibited by ATPase inhibitors (Kim et al., 2013). Consistent with apical constriction playing a role in thyroid outgrowth, incubation of chick thyroid in ATP-containing medium elicits precocious evagination of the bud (Hilfer et al., 1977).

Whereas Rho GTPases including RHOA and RAP1 are known to play roles in promoting apical constriction (Sawyer et al., 2010), available evidence indicates that CDC42 and the PAR6/aPKC complex could be inhibitory to apical constriction. In developing *Drosophila* epithelial tissues, CDC42 antagonizes the action of RHO and reduces apical cell tension (Warner and Longmore, 2009). Consequently, depletion of *Cdc42* results in reduced apical cell area, suggesting an inhibitory role in apical constriction (Georgiou et al., 2008; Warner and Longmore, 2009). Consistent with this, removal of PARD6B from apical cell junctions in cultured mammalian cells also resulted in apical constriction (Ishiuchi and Takeichi, 2011).

However, our study suggests that *Cdc42* might also play a positive role in apical constriction. This may occur either through direct regulation of the apical constriction machinery or as a consequence of its role in promoting apical-basal cell polarity in the endoderm (Fig. 9A,C). Of note is our observation that ZO-1 and F-actin are apically located in CDC42-deficient thyroid buds, suggesting that apical basal polarity is not completely lost in these cells. In *Cdc42* CKO embryos, the normal sub-cellular localization of SHROOM3 is lost, including the apical accumulation at the site of epithelial bending. SHROOM3 is involved in apical constriction in other developmental processes, including the formation of the hinge points in the neural tube that are required for neural tube closure (Hildebrand and Soriano, 1999; McShane et al., 2015) and for invagination of the lens placode (Plageman et al., 2010). In both contexts, the lack of SHROOM3 results in a loss of apical accumulation of Myosin II and a failure of the apical part of the epithelial cells to contract at the point of bending. Previous work has shown that the sub-cellular location of RHOA influences SHROOM3 localization in epithelial cells and knockout of RHOA in the lens pit results in a loss of apical SHROOM3 localization (Plageman et al., 2011). Our data suggest the possibility that CDC42 could fulfill a similar function in localizing SHROOM3 during the formation of the thyroid bud. This could occur either a through specific interaction between SHROOM3 and CDC42 or as a consequence of the general disruption to apical-basal polarity in the foregut endoderm. The mislocalization of SHROOM3 may be crucial to the failure of apical constriction in Cdc42 CKO embryos, as our data also show that SHROOM3 is required for the epithelial bending that allows the thyroid bud to expand ventrally. However, it remains possible that CDC42 and SHROOM3 regulate apical constriction independently.

## MATERIALS AND METHODS

### Mouse strains, genotyping and tamoxifen administration

*Cdc42* conditional (flox) mutant mice (Wu et al., 2006), obtained from Cord Brakebusch (University of Copenhagen), were crossed with *Ubc*-CreERT2 mice (Ruzankina et al., 2007). The resulting double-heterozygous mice were mated with *Cdc42^flox/flox^* mice to generate embryos for analysis. Tamoxifen was prepared at 40 mg/ml in canola oil containing 10% ethanol, stored at 4°C and administered to pregnant female mice at E5.5, E6.5, E7.5 or E8.5 by gavage at a dosage of 0.4 mg/g bodyweight. To assess the efficiency of Cre-mediated deletions at different stages of development, Cre-reporter expression was examined in embryos harvested from *Cdc42^flox/+^; Ubc*-CreERT2 mice that were crossed with ROSA26R mice, containing a *lacZ* transgene that is activated by Cre-mediated excision (Soriano, 1999). Genotyping of tail or yolk sac DNA mice was carried out as previously described (Bildsoe et al., 2009; Wu et al., 2006). Heterozygous *Shroom3^Gt(ROSA)53Sor^* mice (*Shroom3^+/Gt^*) (Hildebrand and Soriano, 1999) were crossed to generate homozygous mutant embryos at E9.0, E9.5, and E10.5. PCR of yolk sac DNA (5′-ATCCTCTGCATGGTCAGGTC, 5′-CGTGGCCTGATTCATTCC, 94°C 30 s; 60°C 30 s; 72°C 30 s; 30 cycles; 315 bp=*Gt* allele) and the presence of neural tube defects (100% penetrance in homozygous mutant embryos) were used to genotype embryos. All animal experimentation was approved by the Children's Medical Research Institute/Children's Hospital Westmead Animal Ethics Committee (project number C314) and carried out in accordance with the Australian Code for the Care and Use of Animals for Scientific Research, or approved by the Ohio State University Office of Responsible Research Practices, and carried out in accordance with all Institutional Animal Care and Use Committee (IACUC) guidelines

### Histology, cryosectioning and staining

Embryos for histological analysis were harvested in PB1 medium, washed in PBS and fixed for at least 16 h in 4% PFA. Specimens were dehydrated through an ethanol series, sectioned and stained with haematoxylin and eosin as previously described (Bildsoe et al., 2009, 2013). Staining for β-galactosidase activity was performed on whole embryos or cryosections as described (Loebel et al., 2012). For cryosectioning, embryos were fixed in 4% PFA and transferred to 20% sucrose/PBS overnight followed by storing in 30% sucrose at 4°C. Embryos were then infiltrated with 30% sucrose:OCT (2:1), frozen in 30% sucrose:OCT (1:2), and cryosectioned at 6-12 μm.

### Immunostaining

Cryosectioned materials were thawed to room temperature, permeabilized in 0.2% Triton X-100/PBS for 5 min and washed 3 times for 5 min in PBT (PBS/0.1% Tween-20). For SHROOM3 staining, antigen retrieval was carried out by incubating in 100 mM Tris-HCl, pH 9.0 heated to 95°C for 45 min. Sections were treated in CAS-block (Life Technologies) for 1-2 h, incubated with primary antibody overnight in CAS-block and washed three times in PBT. Secondary antibody and phalloidin were added in CAS-block for 1-2 h at room temperature, followed by washing in PBT and counterstaining with DAPI or Hoechst 33342 (Sigma, B-2261). Sections were mounted in Fluoromount G and stored at 4°C until imaged.

The following primary antibodies were used: rabbit anti-ZO-1 (Invitrogen 40-2200, 3:1000); rat anti-E-cadherin (Invitrogen 13-1900, 3:1000), rabbit anti-phospho-Myosin Light chain 2 (Cell Signaling 3671, 1:50), rabbit anti-NKX2-1 (Cell Signaling 12373, 1:50), rabbit anti-NKX2-1 (Seven Hills Bioreagents, WRAB-1231, 1:500), rabbit anti-PARD6B (Santa Cruz, 1:500), rabbit anti-SHROOM3 (Genscript custom antibody, epitope CLLEGMRQADIRYVK, 1:1000), rabbit anti-Myosin IIb (Covance PRB445P, 1/1000), rabbit anti-cleaved Caspase 3 (Cell Signaling, 9661, 1/800), rabbit anti-Ki67 (Abcam ab16667, 1:300), Rabbit anti-CDC42 (Cell Signaling, 2466, 1:300). Secondary antibodies used were: Alexa Fluor 546-conjugated goat anti-rabbit (Invitrogen A11010, 3:1000), Alexa Fluor 488-conjugated goat anti-rat (Invitrogen A11006, 3:1000), Alexa Fluor 594-conjugated donkey anti-rabbit (Invitrogen A21207, 3:1000) and Alexa Fluor 594-conjugated goat anti-rabbit (Invitrogen A11008, 1:1000). F-actin was detected with Alexa Fluor 633- or 488-conjugated phalloidin (Invitrogen A22284, 1:200 or A12379, 1:1000, respectively).

### Imaging and image analysis

Whole mount and histological sections were imaged using SPOT cameras and software. Confocal images were acquired on a Leica SP5 confocal microscope. Brightness, contrast and white-balance adjustments were carried out using Photoshop or Pixelmator. Image analysis was performed using ImageJ. Nuclear height:width ratios were calculated from measurements of the length of the nucleus perpendicular and parallel to the apical surface respectively. Relative fluorescence intensity of immunostaining, computed as an apical:basal ratio, was determined by measuring the mean pixel intensity along lines drawn at the apical and basal aspect of 10-20 cells per section. Intensity profiles were plotted for each channel of representative images along a line through the section as indicated in the figures. Individual channel profiles were combined into a single plot.
